# Supplementary material for: Sustained-input switches for transcription factors and microRNAs are central building blocks of eukaryotic gene circuits
Source: Genome Biol. 2013 Aug 23;14(8):R85. doi: 10.1186/gb-2013-14-8-r85 (PMC4054853; doi:10.1186/gb-2013-14-8-r85)
Supplement: Additional file 5 — HTML Browsable Motif Output. Zipped folder containing all WaRSwap and FANMOD motif output, viewable in a web browser. [file gb-2013-14-8-r85-S5.ZIP › HTML_browsable_motif_output/WaRSwap_all_tair9/sigs_WSWRSLDraw-2000_all.pvals.heatmaps.html/motif_id_38_001101002_tftype_all_upstream_-1000_0.html]

```
BG_MODEL = Rnew2
MOTIF_ID = 38_001101002
TF_TYPE = all
UPSTREAM = -1000_0


PVals
FN_0.2	FN_0.4	FN_0.6	FN_0.8
dg_60.genes	0.997	0.256	0	0.017
dg_70.genes	0.999	0.228	0	0.018
dg_80.genes	0.997	0.247	0	0.012

ZScores
FN_0.2	FN_0.4	FN_0.6	FN_0.8
dg_60.genes	-2.698	0.689	5.31	2.268
dg_70.genes	-2.739	0.772	5.35	2.202
dg_80.genes	-2.674	0.707	5.303	2.344

StDevs
FN_0.2	FN_0.4	FN_0.6	FN_0.8
dg_60.genes	974490.019	163867.007	30217.303	4772.851
dg_70.genes	971946.914	160274.372	30094.556	4912.239
dg_80.genes	973955.269	160712.271	30227.765	4662.869
```
